# Bandwidth Enhancement and Generation of CP of Yagi-Uda-Shape Feed on a Rectangular DRA for 5G Applications

**DOI:** 10.3390/mi13111913

**Published:** 2022-11-04

**Authors:** Inam Bari, Javed Iqbal, Haider Ali, Abdul Rauf, Muhammad Bilal, Naveed Jan, Usman Illahi, Muhammad Arif, Muhammad Amir Khan, Rania M. Ghoniem

**Affiliations:** 1 Systems Engineering Department, Military Technological College, Muscat 111, Oman; 2 Electrical Engineering Department, FET, Gomal University, Dera Ismail Khan 29050, KPK, Pakistan; 3 Department of Electrical and Electronics Engineering Technology, University of Technology Nowshera, Nowshera 24100, Pakistan; 4 Department of Electrical Engineering, National University of Sciences and Technology, H-12, Islamabad 44000, Pakistan; 5 Department of Information Engineering Technology, University of Technology Nowshera, Nowshera 24100, Pakistan; 6 Center for Advanced Studies in Energy, University of Engineering and Technology, Peshawar 25000, Pakistan; 7 Department of Computer Science, COMSATS University, Islamabad-Abbottabad Campus, Abbottabad 22060, Pakistan; 8 Department of Information Technology, College of Computer and Information Sciences, Princess Nourah bint Abdulrahman University, P.O. Box 84428, Riyadh 11671, Saudi Arabia

**Keywords:** band singly fed, circular polarization, flat surface  metal strip, dielectric resonator antenna (DRA), Yagi-Uda, 5G N.R.

## Abstract

A wideband circularly polarized rectangular dielectric resonator antenna (DRA) fed by a single feeding mechanism has been studied theoretically and experimentally. The purpose of the study is to determine how adding a parasitic strip next to the flat surface metallic feed would affect various far- and near-field antenna characteristics. Initially, the basic antenna design, i.e., the T-shape feed known as antenna A, produced a 4.81% impedance matching bandwidth (|S_11_| −10 dB). Due to the narrow and undesirable results of the initial antenna design, antenna-A was updated to the antenna-B design, i.e., Yagi-Uda. The antenna-B produced a decent result (7.89% S_11_) as compared to antenna-A but still needed the bandwidth widened, for this, a parasitic patch was introduced next to the Yagi-Uda antenna on the rectangular DRA at an optimized location to further improve the results. This arrangement produced circular polarization (CP) waves spanning a broad bandwidth of 28.21% (3.59–3.44 GHz) and a broad impedance |S_11_| bandwidth of around 29.74% (3.71–3.62 GHz). These findings show that, in addition to producing CP, parasite patches also cause the return loss to rise by a factor of almost three times when compared to results obtained with the Yagi-Uda-shape feed alone. Computer simulation technology was used for the simulation (CST-2017). The planned antenna geometry prototype was fabricated and measured. Performance indicators show that the suggested antenna is a good fit for 5G applications. The simulated outcomes and measurements match up reasonably.

## 1. Introduction

The DRA is a unique kind of antenna that utilizes the radiating modes of a dielectric resonator (DR). In terms of variety of material and shape, the DRA offers a higher margin of flexibility and adaptability than 1-D linear and 2-D planar antennae. Other desirable qualities of DRA include its compact size, minimal ohmic loss, and simplicity of excitation. The DRA is viewed as a viable contender for the next generation of wireless communications because of these important properties [1].

Circularly polarized DRAs have gained attention recently due to features such as being less affected by atmospheric conditions, more capable of mitigating polarization mismatch, and in particular not being sensitive to how the transmitting and receiving antennas are oriented. Initially, the primary research focus was on linearly polarized (LP) antennas. For satellite communication and navigation systems, global positioning systems, and radio frequency identification systems, circularly polarized DRAs are preferred [2,3,4,5,6,7,8,9,10,11,12,13,14,15]. Although these techniques make the design more intricate and sensitive, a wider bandwidth has been attained through such design modifications. Additionally, perforation DRA delicate cutting is a difficult task; however, by inserting the parasitic patch, these issues may be resolved without sacrificing the DR’s size and form.

In general, there are three different ways to generate the CP fields of a DRA: single feed, dual feed, and quadrature feeds [2,3]. Wider impedance matching (S_11_) and axial ratio (3 dB) bandwidths may often be achieved using quadrature or dual-feed mechanisms; as a result, power dividers or external hybrid couplers are employed, unavoidably increasing the total size, complication, and cost of the antenna system. In parallel, the feeding technique, such as a single probe/flat surface metal strip, uses a specific feeding structure and typically has an extremely simple and easy feeding network; however, it has the shortcoming of having a small impedance matching bandwidth along with a narrow 3 dB axial ratio of less than 10%. This technique generates circularly polarized waves primarily through exciting the dielectric resonator geometry. This is why there has been a lot of focus on increasing the bandwidth of circularly polarized DRAs energizing through a singly fed mechanism [4].

The impedance matching and 3 dB axial ratio (AR) bandwidths of single-feed CP DRAs have reportedly increased using a variety of approaches in the literature, including a straightforward method that utilizes [5,6] DRs. The entire strategy will result in bandwidth improvement by configuring the DRs to operate at close frequencies.

For instance, a 22% bandwidth may be achieved by just arranging two rectangular DRs in a stair-shaped DRA [7], although such a method makes the geometry a little more challenging.

Additionally, a singly fed differentially stimulated hollow rectangle DRA has been described in [8]. The hybrid coupler was utilized to excite the device and the two flat surface strips were employed to transmit the differential signal. With that method, the AR bandwidth was 11.8%. On the other hand, frequency reconfigurable antennas [9,10] are compact in size, use the single feed excitation mechanism, and have better results in near-field characteristics but they are not circularly polarized. For Sub-6 GHz and WLAN, application compact size antennae are utilized [11]; however, they have complex feeding techniques which make the design a bit complicated. A novel method that uses a parasitic strip or patch in conjunction with a flat surface strip to feed the DRA has just been proposed in [12]. The parasitic strip causes two nearly degenerate orthogonal modes to be activated, which result in CP fields and disturb the DRA fields. A single feed may be used to apply the new approach to a traditional DRA. The AR bandwidth was increased from 6.5% to 20% by adding a patch (parasitic) within a round/circular loop antenna [13]. The observed CP bandwidth of 7.13%, which was found to be three times broader than without the parasitic element, was produced by a driven open half-loop conducting metal strip with a parasitic patch in the following work [14].

Moreover, thin flat surface conducting strips have been installed on cylindrical DRAs [15] for both parasitic and feeding purposes. As a result, the return loss has increased from 5.1% to 11.5% while also producing an AR. Cutting the corners/edge of the DRA and placing a flat surface parasitic strip nearby is another method to increase return loss and create CP; this method dramatically increases S_11_ to 49.7% and the axial ratio bandwidth to 11.7% [16].

In the last decade, a lot of work has been done to design antennas that can cover 5G applications, such as in [17]; here, the array has been used to achieve a broader bandwidth and covers 5G applications. However, such antennae have a complex design and are linearly polarized, while in parallel, [18] have a simple geometry design but have the drawback of a narrow 10-dB impedance bandwidth. In [19,20,21], the authors achieve a reasonable bandwidth in both return loss and axial ratio but have a complicated geometry design. Microstrip patch antennae are used in [22], which have the disadvantage of metallic loss at higher frequencies. Dipole antennas are also used for 5G applications [23,24] but the performance of these antennas is too good when used as an array or MIMO.

In order to overcome all the above-mentioned issues, a broadband CP DRA is presented in this article, where a parasitic patch is added at an ideal distance next to the Yagi-Uda-shaped flat surface metal strip to solve the narrow bandwidth issue, as discussed before with antenna-A and antenna-B. The findings are shown here with the help a of simulating tool, i.e., CST Microwave studio [25], which generated broader (|S_11_| −10 dB) impedance matching and 3 dB axial ratio bandwidths of 29.74% and 28.01%, correspondingly, in addition to the improved gain of 6.65 dBic. The broadband CP DRA prototype is constructed, and measurements show good agreement between real-world and computer simulation findings. The suggested DRA’s working spectrum spans the frequency range of 3.67–4.60 GHz, making it suitable for 5G applications

The structure of the article is as follows. The configuration of the antenna components is presented in Section 2. This is followed by a discussion of the theoretical implications, working principle, bandwidth augmentation, production of CP waves, and design requirements. The experimental results of the produced antenna are shown in Section 3. The findings are drawn in Section 4.

## 2. Antenna Design and Analysis

In addition to discussing the dimensions of the rectangular DRA with and without a parasitic patch, this subsection also explains the CST’s operating principles and simulated outcomes.

### 2.1. Antenna Configuration

**Figure 1:**
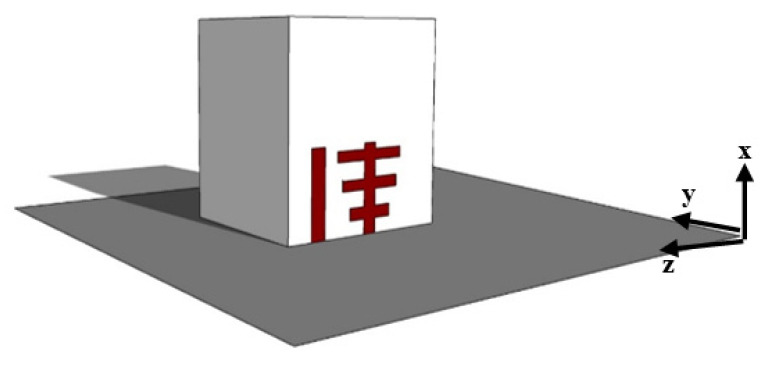
Three-dimensional view of the proposed singly fed Yagi-Uda shape RDRA.

**Figure 2:**
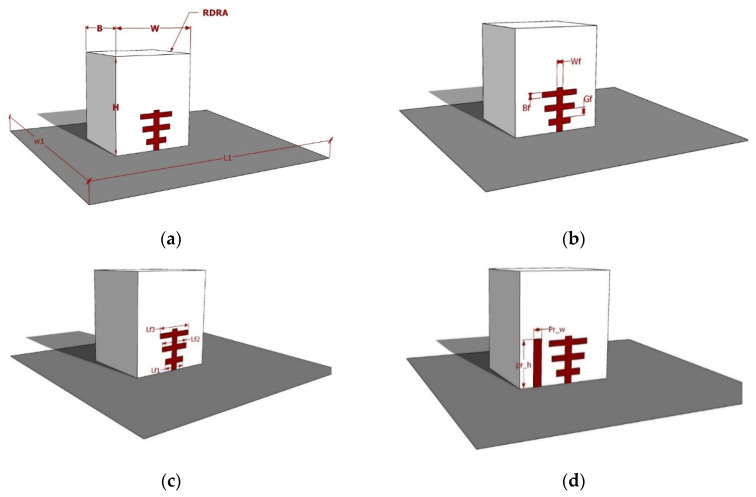
Dimension of the Proposed Yagi-Uda RDRA antenna: (**a**) Dimension of the RDRA; (**b**–**d**) dimension of the Yagi-Uda Feed structure and parasitic element.

According to Figure 1, the proposed singly fed broadband with a circularly polarized DRA comprises a rectangular DRA, a Yagi-Uda-shaped flat surface feeding strip, a parasitic patch, and a PEC ground plane. As depicted in Figure 2, the rectangular DRA’s dimensions are retained the same as in [18], where they are H = 26.1 mm in height, B = 25.4 mm in width, and W = 14.3 mm in depth. Additionally, the material ECCOSTOCK HIK, with a relative permittivity of 9.8 and a tanδ of 0.002, is used to build the RDRA. A novel-shaped flat surface metal strip was adhered to the DRA face using glue (quick fix). Flat surface metal strips, which are constructed of four separate cut strips, excite the antenna and form the Yagi-Uda feed structure.

A finite integration method (FIT) based simulation tool, called CST MWS, was used for providing the best parameters for the proposed wideband RDRA feed. The optimal parameter for the strips is then displayed in Table 1. The 35 × 35 cm^2^ of square PEC ground has been utilized. A rectangular DRA was placed in the center of the PEC ground plane.

### 2.2. Evolution of Wideband RRDA Antenna

**Figure 3:**
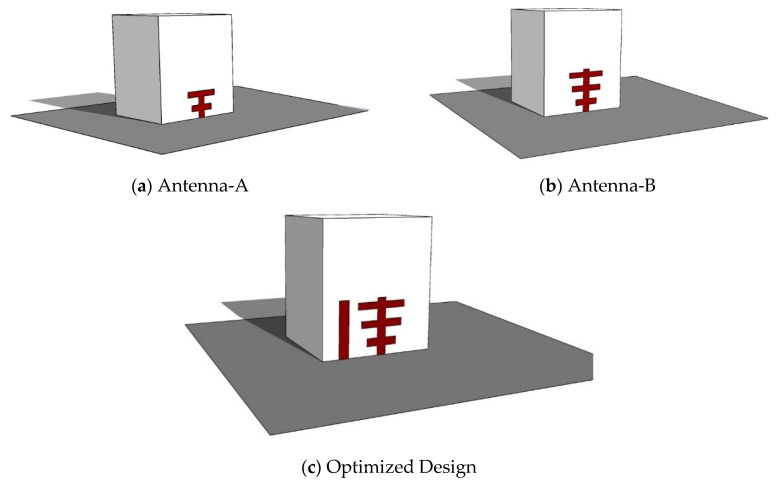
Evolution of the RDRA wideband antenna (3-D View) (**a**) Antenna-A, RDRA with the T-shape feed; (**b**) antenna-2, RDRA with a novel flat surface metal strip (Yagi-Uda); (**c**) desired wideband RDRA with the parasitic patch.

The proposed wideband RDRA antenna’s development and construction are shown in Figure 3. The layout in Figure 3a depicts the antenna-A configuration, which consists of three metal strips to have a T-shaped feed and the said feed is excited by a 50 Ω coaxial probe.

In step 2, antenna-B has been developed from antenna-A. The new feed of the antenna-B is designed by updating the feed from step -1 using four flat surface metal strips which results in creating a Yagi-Uda shape feed, as seen in Figure 2b. Finally, the proposed wideband RDRA is created by adding a parasitic patch at an ideal place beside the Yagi-Uda feed, as depicted in Figure 3.

### 2.3. Simulation of Antenna-A Antenna-B Design, Analysis, and Discussion

**Figure 4:**
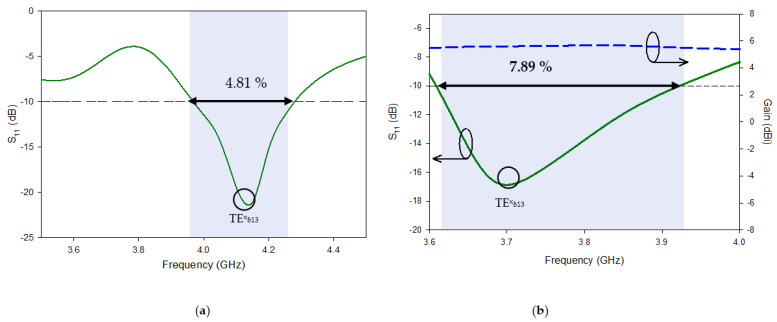
Simulated S_11_ and Gain of (**a**) antenna-A and (**b**) antenna-B.

This subsection explains the antenna-A simulation’s outcome. Figure 4 shows that antenna-A has a narrow impedance matching bandwidth (|S_11_| −10 dB) of just 4.81% (3.96–4.28 GHz) and a resonance frequency of 4.15 GHz. The resonance frequency can be approximately calculated by [25].

(1) $$f_{0}=\frac{1.8412c}{2\pi L_{p}Ɛr^{\frac{1}{2}}}$$

where *c* is the speed of light, Ɛr = 9.8 and *Lp* is the length of the flat surface metal strip. To suppress the reflection from the edges of the dielectric cover (flat surface strip), the shape of the dielectric cover is tapered linearly at the same rate as Yagi. Generally, the directivity of Yagi is inversely proportional to the square of effective wavelength [26]. The relationship between directivity and effective wavelength is described in the following equations:

(2) $$D=4\pi {\frac{L_{p}}{\lambda_{eff}}}^{2}$$

(3) $$\lambda_{eff}=\frac{\lambda o}{Ɛl^{\frac{1}{2}}}$$

where Ɛl = is the effective dielectric constant of dielectric with a Yagi-shaped flat surface metal strip.

It should be noted that the antenna is not only narrow-banded but also a linearly polarized antenna because the T-shaped feed only excites a single higher-order mode, TE_x13_, inside the DR, whereas it is necessary to excite an orthogonal mode pair. It is obvious that the results from antenna-A are undesirable and cannot be used for 5G applications because of the narrow band. In order to overcome this issue some modifications have been performed on antenna-A which leads the design to the Yagi-Uda shape, this design is named antenna-B. Through simulation results, it has been observed that the results are not improving much, with S_11_ just improving from 4.81% to 7.94%. Poor return loss is due to poor impedance matching, which is due to the large impedance when the periodic dipole strips work in their fundamental TExδ13 mode. With an irregular gradient structure [27], the central resonant frequency shifts to the right from 4.15 GHz to 3.7 GHz. The shift in resonance frequency is used to calculate by ∆Ɛe =0.012 and Ɛe = 1.506 by Equations (4) and (5). According to [28], the effective permittivity Ɛe_0_ of the dielectric material of DRA can be calculated by

(4) $$Ɛe_{0}=\frac{Ɛr1-1}{2}\frac{k2}{k1}$$

Here,

(5) $$K_{1}\;=\frac{l}{l+2s},\;k_{2}\;=e^{\frac{-\pi s}{2h}}$$

where Ɛ*r*1 is the permittivity of dielectric material while where Ɛe0 effective dielectric constant of Yagi; l is the length of the active dipole; s is the gap between dipole arms; ∆Ɛe is the change in effective dielectric constant.

### 2.4. Incorporating Parasitic Patch

So far, the return loss and gain results are not significant. In order to improve the impedance-matching bandwidth and generate CP waves, the parasitic patch is introduced at an optimized location beside the flat surface metal strip. Incorporating the parasitic patch not only widens the impedance matching of the antenna but is also responsible for the CP waves. The initial dimension of the parasitic patch can be approximately determined with the help of the following equation:

(6) $$\frac{\pi \gamma }{180}=\frac{Lp}{\left[C_{o}f_{o}\right]\sqrt{\frac{Ɛe+1}{2}}}$$

where *γ* = 90°.

### 2.5. Working Principle, Generation of CP, Simulation Results of Design, Analysis, and Discussion

**Figure 5:**
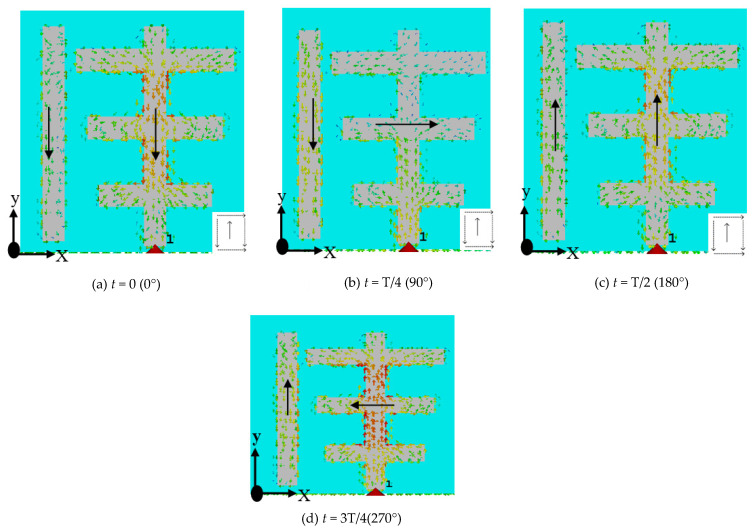
Distribution of simulated current at 3.75 GHz at: (**a**) *t* = 0 (0°), (**b**) *t* = T/4 (90°), (**c**) *t* = T/2(180°), and (**d**) *t* = 3T/4(270°).

The new design after implementing the parasitic patch forms a sequential rotation arrangement resulting in an improvement in impedance match bandwidth and generation of CP [29,30]. The simulated surface current distributions of the antenna at 3.74 GHz (Minimum of S_11_), as shown in Figure 5. This may be used to understand the impact of parasitic patches on the electric field. The y-component of the current on the parasitic patch and that of the induced current on the Yagi-Uda patches are orienting in the same direction, as can be seen in Figure 5a,c; although their x-components are orienting in opposite directions, as can be seen in Figure 5b,d.

Furthermore, it is not difficult to conclude that the induced current on the parasitic patches decreases E_θ_ but increases E_Ҩ_ given that the x-component contributes to E_θ_ while the y-component contributes to E_Ҩ_. To further study the broadband CP property of the designed antenna, the current concentrates on the novel shape flat surface metal strip at *t* = 0, *t* = T/4, *t* = T/2, and at *t* = 3T/4. Moreover, it is seen that the composite current surface currents on the novel feed are orthogonal at 0° and 90°, which provides the required condition for CP generation. Thus, the antenna owns CP performance due to the orthogonal current direction. Surface current distribution at 180° and 270° opposes the direction of the currents at 0° and 90°. In addition, the current distributions sequentially rotate in an anticlockwise direction, so RHCP performance is exhibited.

### 2.6. Parasitic Strip Optimization

**Figure 6:**
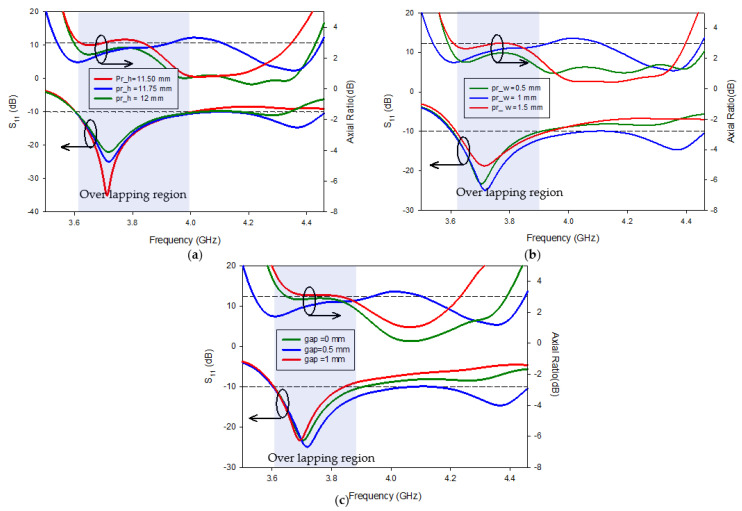
Parametric examination of return loss and axial ratio for various: (**a**) Height, (**b**) width, and (**c**) positions of the parasitic strip.

Figure 6a–c illustrate the variation in the height (Pr_h), the gap between the parasitic patch and the Yagi-Uda strip, and the width (Pr_w) of the parasitic strip, respectively, together with the difference in the impedance-matching characteristic |S_11_| and 3 dB axial ratio. Figure 6a shows that the resonant frequency does not vary as the height of the parasitic patch changes but the bandwidth S_11_ decreases by −10 dB and exceeds −10 dB at about 4 GHz. This is brought about by the parasitic strip’s modification of the electrical length of RDRA in the y-direction.

Axial ratio fluctuation is shown in Figure 6a in a similar way. The height of the parasitic patch was discovered to enhance the axial ratio bandwidth; however, the overlapping region is found to be small due to the S_11_ bandwidth. Figure 6b,c exhibit, respectively, the variation in the return loss characteristic and the axial ratio bandwidth due to changes in the breadth and gap between the parasitic patch and the feed. The width and gap of the parasitic strip have slight effects on the axial ratio bandwidth, even when the other parameters (|S_11_|) are simultaneously changing. This is because of the parasitic strip that loaded DR modes, such as TExδ13 and TEy1δ3, in the lower band. Parametric studies enable the determination of the parasitic patch’s optimized parameters. Likewise, the proposed antenna offers a simple axial ratio and impedance adjustment.

## 3. Simulated Result of the Proposed Antenna

This segment introduces a bandwidth improvement method and CP wave production. It is noted that inserting the parasitic patch helps in creating extra resonant frequency, which in turn helps in reaching a broader bandwidth [31]. A parasitic patch of flat surface metal strip is positioned next to the Yagi-Uda-shaped feed at an optimal gap. This caused two orthogonal degenerate modes to emerge, nearly one on each side of antenna-A’s return loss (S_11_) values.

**Figure 7:**
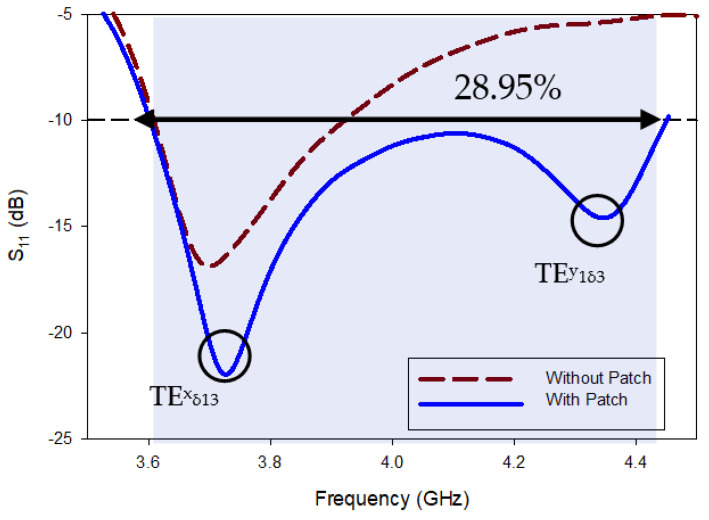
Analysis of simulated return loss with/without patch.

**Figure 8:**
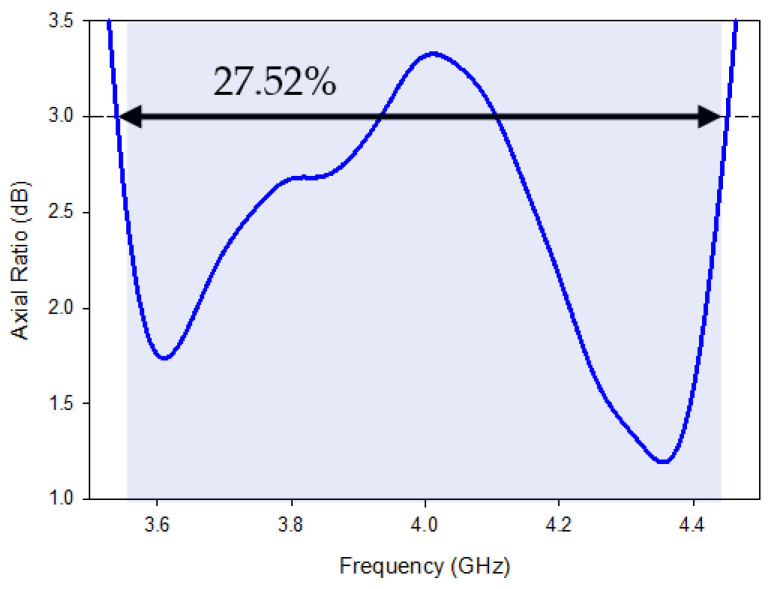
Axial ratio with parasitic patch.

**Figure 9:**
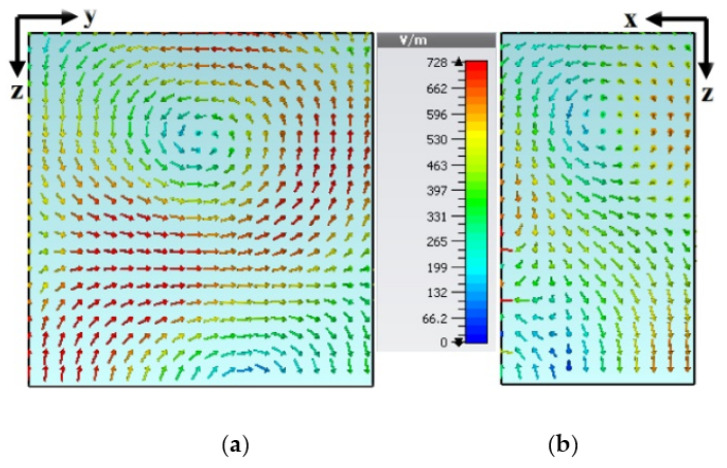
Electric fields: (**a**) TExδ13 at 3.74 GH and (**b**) TEy1δ3 at 4.4 GHz.

**Figure 10:**
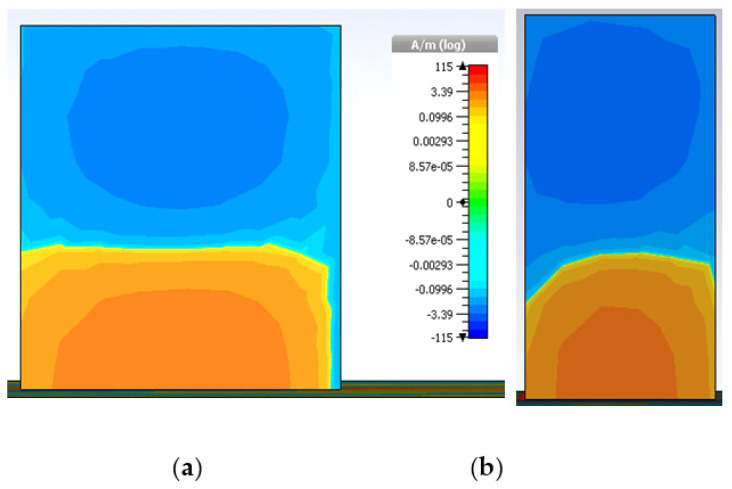
Magnetic fields: (**a**) TExδ13 at 3.74 GHz and (**b**) TEy1δ3 at 4.4 GHz.

The impedance-matching bandwidth graph in Figure 7 shows the increase in bandwidth caused by switching from antenna-B to the predicted DRA. When there is no parasitic strip, this behavior makes it clear that there is just one mode. However, when the patch is added beside the Yagi-Uda-shaped single feed, a 28.95% impedance bandwidth was attained, in the range from 3.59 GHz to 4.40 GHz. It has been determined that the proposed antenna’s bandwidth is around three times greater than that of antenna B. In parallel, two new modes TExδ13 and TEy1δ3 are created at the same 3.72 GHz and 4.37 GHz frequencies, as in [32]. Additionally, as shown in Figure 8, the suggested antenna’s lowest axial ratio of 3.75 GHz is virtually between the degenerated orthogonal modes. Thus, the suggested feeding arrangement of the parasitic patch has met the requirement for the creation of circular polarization. [33]. The parasitic patch-achieved simulated 3 dB axial ratio is 27.52% (3.52–4.45 GHz). Within the simulated bandwidth for impedance matching, the full axial ratio band completely disappears. Figure 9 depicts the simulated electric field distributions to help with understanding the parasitic patch’s basic working principle. Figure 10 illustrates the one-and-a-half field variation that the Yagi-Uda-field vectors make. In order to support the E-field results, the magnetic field distributions also showed higher-order modes.

## 4. Measured Results and Discussion

A prototype of the suggested CP DRA has been constructed and measured for validation purposes. The antenna is made up of a rectangle DR constructed using ECCOSTOCK HIK with a loss tangent tan = 0.002 and a dielectric constant of 10. The DRA is excited using a Yagi-Udi-shaped flat surface metal strip; nowadays, a flat surface strip can take the place of the conventional probe feed. In addition to having problems with air gaps that lead to frequency inconsistencies, coaxial feeding also makes it simple to connect to the coaxial feed line of the SMA connection [34]. Additionally, the parasitic strip was composed of copper tape with conductive glue that easily adhered to the DRA wall.

**Figure 11:**
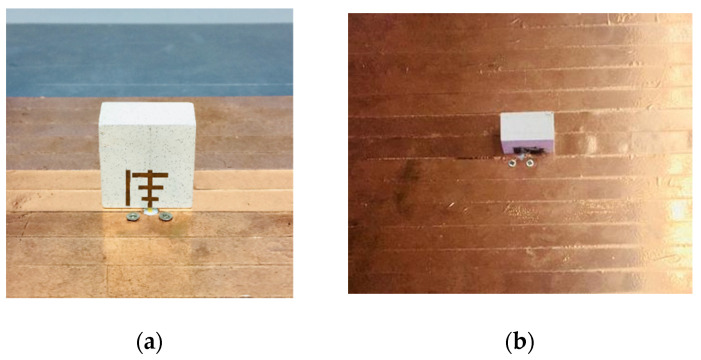
Photo of fabricated singly fed RDRA with parasitic patch: (**a**) front view and (**b**) back view.

Figure 11 shows a shot of a constructed singly fed RDRA with a parasitic strip in the front and the back view. A KEYSIGHT N5234A network analyzer (10 MHz–43.5 GHz) was used to test the input impedance (|S_11_| −10 dB), in parallel an anechoic chamber was utilized to evaluate the far-field characteristics including CP, radiation pattern, and gain (Atenlab OTA-500).

**Figure 12:**
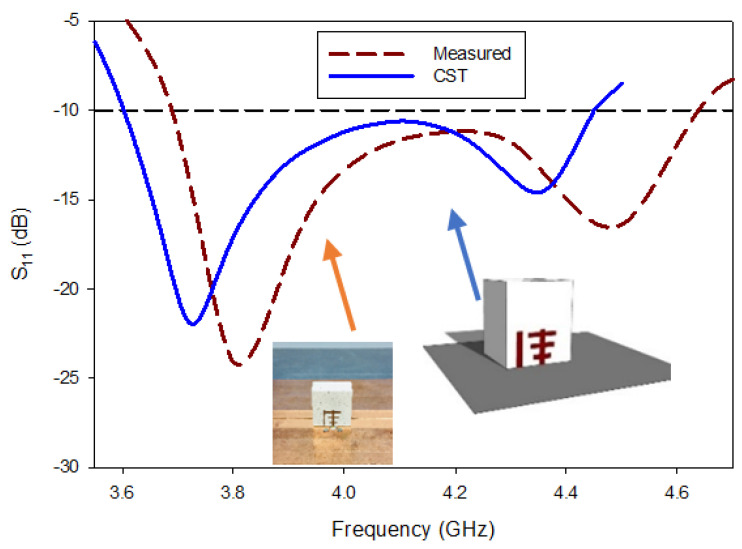
Comparison of return loss of fabricated and simulated RDRA.

**Figure 13:**
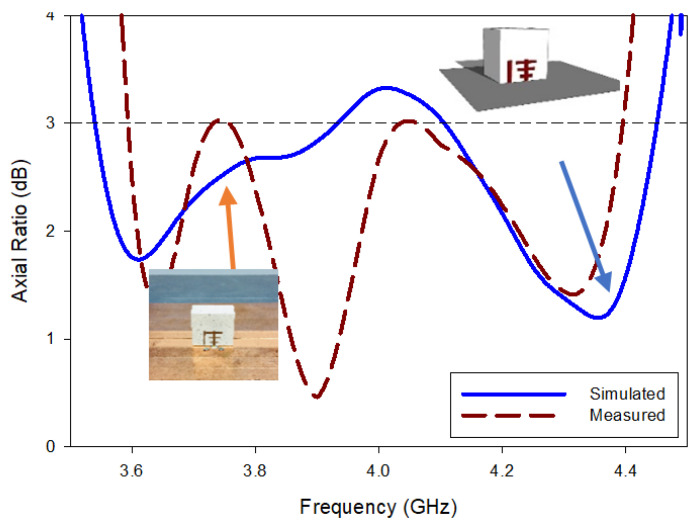
Assessment of the AR of fabricated and simulated RDRA.

**Figure 14:**
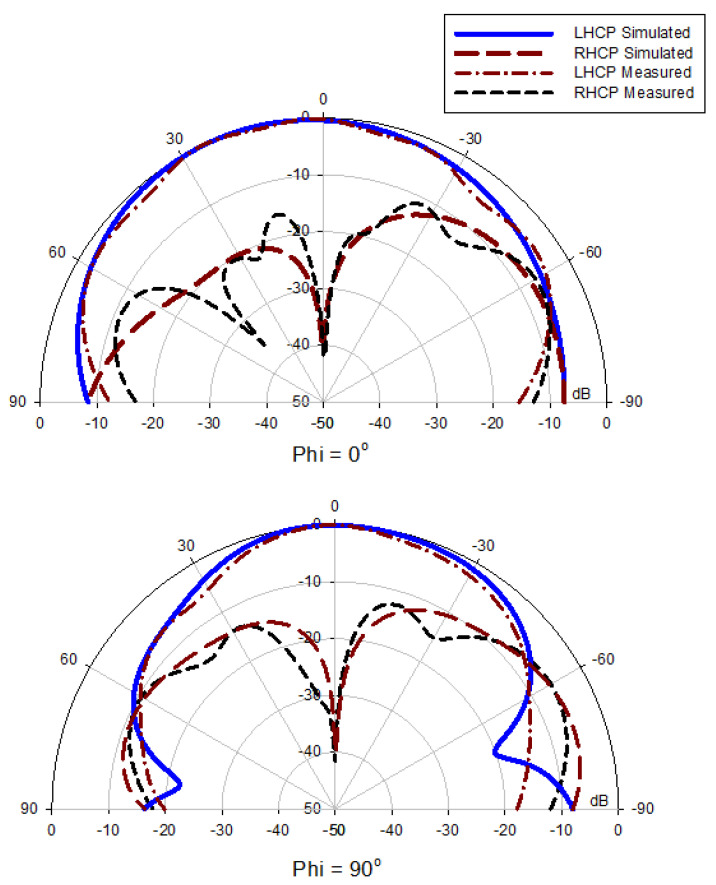
Radiation patterns of the proposed antenna at 3.7 GHz.

**Figure 15:**
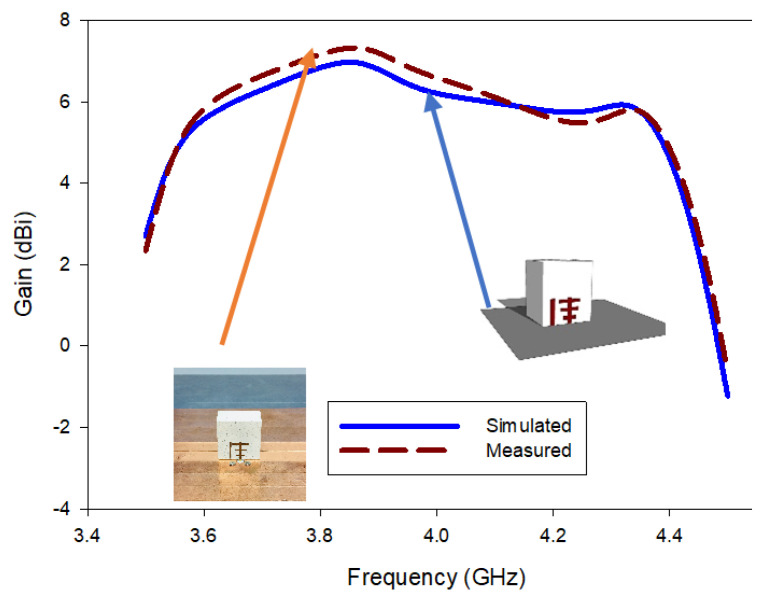
Gain of the proposed antenna.

All the near- and far-field parameters of the proposed antenna, i.e., simulated and fabricating reflection coefficients (Figure 12), AR (Figure 13), radiation pattern (Figure 14), and boresight gains (Figure 15) are shown. Theoretical and experimental findings are reasonably in accord and the tiny discrepancy is mainly triggered by measurement and fabrication flaws. The prototype’s modeled and experimental wide input impedance bandwidths are 28.95% (3.59–4.40 GHz) and 29.74% (3.71–3.62 GHz), respectively, as depicted in Figure 12. The simulated and measured 3 dB AR bandwidths are 27.52% and 28.01%, respectively, as shown in Figure 13.

In addition, they are around three times wider than those of the rectangular DRA design with a Yagi-Uda-shape feed alone. The experimental return loss (S_11_) and CP (3 dB) bandwidths roughly overlap with one another. Figure 14 shows the constructed DRA’s radiation patterns at a minimal axial ratio frequency of 3.7 GHz. Broadside patterns are obtained in both planes, i.e., phi = 0° and phi = 90°, respectively, as expected. It is a good left-hand circularly polarized antenna because the left-hand circularly polarized fields for both planes are more than 26 dB stronger in the direction of the boresight than their right-hand equivalents. On the other hand, as shown in Figure 15, the prototype DRA provides an acceptable calculated and examined boresight gain of 6.49 dBic and 6.71 dBic, respectively, throughout the full desired band.

In order to validate the results further, the mode frequencies achieved by CST closely match those predicted by the Dielectric Waveguide Model (DWM) [35], as provided in Table 2. Additionally, the comparison of simulated and measured results of the proposed antenna in tabulated form is depicted in Table 3. Table 4 contains a quick comparison of the previously mentioned designs and the projected broadband circularly polarized DRA based on S_11_ bandwidth and CP generation techniques.

## 5. Conclusions

In this research, a modified novel feed CP DRA was examined. Primarily, it has been found that the initial design (antenna-A) produced a narrow impedance-matching bandwidth. In order to broaden the return loss, some modifications were performed on the original design (antenna-B). Such changes enhanced the 10 dB bandwidth but still, the desired results (circular polarization) were not achieved. For this, one of the renowned techniques was used to generate circular polarization, i.e., a parasitic patch. The optimized parameter of the parasitic patch was achieved by running a parametric sweep. After obtaining the optimized dimension and position, a parasitic metallic patch was placed next to the feeding strip on the DRA wall. As the parasitic patch generated additional dips, this result caused the appearance of two orthogonal degenerate modes that produce CP waves and dramatically increased the impedance-matching bandwidth from 7% to 28%. The parasitic patch offers stable links and overcomes the limited ARBWs of single-fed antennas. The antenna operates at 3.8 GHz and 3.7–4.6 GHz with 10 dB impedance bandwidths of 28.01% for full-duplex operation. The measured 3 dB AR bandwidth was 28.01%. Reasonable consistency was found between the measured and simulated results. The proposed DRA would be a promising candidate for 5G applications.

## Figures and Tables

**Table 1:** Best estimates of proposed wideband RDRA and feeds dimensions.

Element.	Parameter	Dimension (mm)
PEC Ground Plane	L_1_, W_1_	35, 35
RDRA	H, W, B	26.1, 14.3, 25.4
Yagi-Uda Feed	Wf, Bf, Gf, Lf1, Lf2, Lf3	1, 1, 2, 3, 5, 7
Parasitic Strip	Pr_h, Pr_w	11.75, 1.00

**Table 2:** Analysis of the measured, calculated, and projected DRA frequencies for TE^x^_δ13_ and TE^y^_1δ3_ modes.

Resonant Modes	Fabricated Resonant Frequency	Simulated Resonant Frequency (CST)	Anticipated Resonant Frequency DWM
	*f*_MEA_ (GHz)	*f*_CST_ (GHz)	*f*_DWM_ (GHz)
TE^x^_δ13_	3.8	3.73	3.89
TE^y^_1δ3_	4.5	4.46	4.53

**Table 3:** Analysis of measured and simulated results.

CST	Frequency Range (S_11_) (GHz)	Return Loss (%)	Frequency Range (3 dB) (GHz)	Axial Ratio BW (%)	Gain (dBic)
	3.6–4.45	~28.95	3.59–4.40	27.52	6.64
Measured	3.71–4.62	~29.74	3.59–3.44	28.01	6.50

**Table 4:** Analysis with earlier published work in literature.

Ref	Feeding Mechanism	Wideband Mechanism	Impedance BW GHz,(%)	Overlapping BW %	Gain Max (dBi)	Structure
[14]	Singly fed microstrip, coupled cross slot	Square DRA + 4 parasitic vertical plates	2.2–3.6 (46.9)	49.5	4.7	Complicated
[4]	Flat surface strip	RDRA + parasitic patch	2.95–3.65 (13)	6	5	Simple
[8]	Flat surface strip	CDRA + Parasitic patch	2.31–2.4 (11.5)	3	4.02	Simple
[15]	Coaxial probe feed	RDRA (cutting edge) + floating parasitic strip	6.57–12.18 (59.8)	10.6	4.86	Simple
[20]	Cross slot	RDRD + multilayer	9.5–12.5 (21)	9.5	11	Complicated
Proposed work	Singly fed flat surface strip	RDRA + parasitic patch	3.6–4.45 (29.74)	28.01	6.5	Simple

## Data Availability

All the data have been included in study.
